# Association of Chemical Aggregates and Fungal Moieties Affecting Native Environmental Films

**DOI:** 10.1021/acsenvironau.2c00004

**Published:** 2022-04-14

**Authors:** Jessica L DeYoung, Scott K. Shaw

**Affiliations:** Chemistry Department, University of Iowa, Iowa City, Iowa 52242, United States

**Keywords:** Grime, fungi, aggregates, microbiotic nutrients, carbon recycling, environmental films

## Abstract

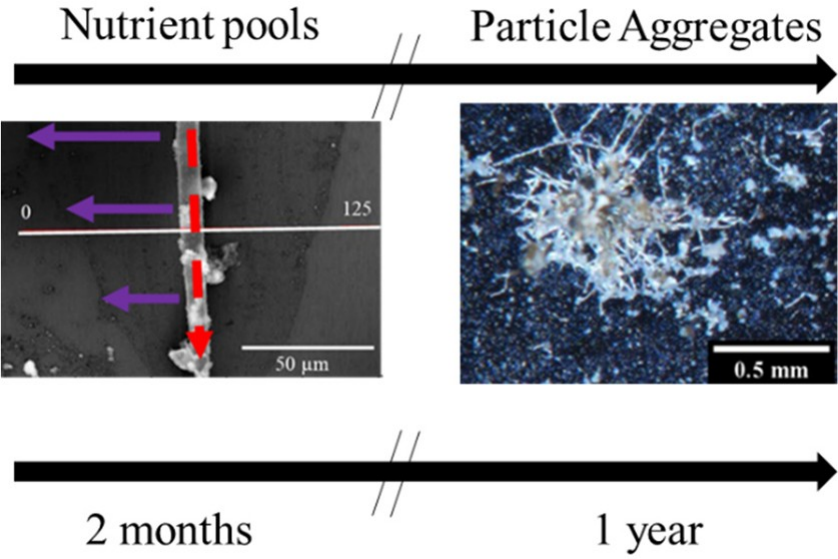

Fungi are prevalent microorganisms in environmental films. Their impacts on the film chemical environment and morphology remains poorly defined. Here we present microscopic and chemical analyses fungi impacts to environmental films over long- and short-time scales. We report bulk properties of films accumulated for 2 months (February and March 2019) and 12 months to contrast short and longer-term effects. Bright field microscopy results show that fungi and fungal-associated aggregates cover close to 14% of the surface after 12 months and include significant numbers of large (tens to hundreds of μm in diameter) particles aggregated with fungal colonies. Data acquired for films accumulated over shorter times (2 months) suggest mechanisms that contribute to these longer-term effects. This is important because the film’s exposed surface will determine what additional material will accumulate over the ensuing weeks or months. A combination of scanning electron microscopy and energy dispersive X-ray spectroscopy provides spatially resolved maps of fugal hypha and nearby elements of interest. We also identify a “nutrient pool” associated with the fungal hypha which extend orthogonally to the growth direction to ca. 50 μm distances. We conclude that fungi have both short-term and long-term effects on the chemistry and morphology of environmental film surfaces. In short, the presence (or absence) of fungi will significantly alter the films’ evolution and should be considered when analyzing environmental film impacts on local processes.

## Introduction

Accumulations on impervious outdoor surfaces, known as environmental films (EFs), directly impact the environment by adsorbing and changing existing atmospheric species.^1−3^ The film material is mostly inorganic (50– 90%) with the balance being organic or unidentified.^4−6^ Elemental and molecular compositions have primarily been identified by methods that require dissolution and overlook the materials’ physical forms.^1,7,8^ Studying EF physical forms has allowed the identification and characterization of diverse and thriving populations of fungal and bacterial species.^9^ The purpose of this work is to evaluate how these microorganisms change the chemistry and morphology of the surface, creating microenvironments as they grow.^10^

Initially clean surfaces collect EFs, accumulating carbonaceous material and micronutrients, including fixed nitrogen species, to facilitate microorganism growth. A generic form of fungal growth is extension from a center via filamentous branches called hyphae.^11^ The growth and extension of hyphae depend on environmental conditions such as temperature, humidity, and available nutrients.^12^ A few studies have reported seasonal variations in EF chemical composition^7,13^ which are relevant here because many EFs will have seasonally limited nutrient supplies. One seasonal trend includes higher fixed nitrate concentration in the summer months^7^ which is important because fixed nitrogen availability and carbon–nitrogen ratios are important factors for fungal growth.^14,15^ Generally, EF fixed nitrate concentrations are highest in the summer months. When conditions for growth are not ideal, some fungi can also source nutrients from recycling nitrogen containing minerals.^16^ Alternative nitrogen sources may include organic species in the EF such as those that our group have previously observed.^9^

The presence and growth of fungi will change the chemical profile of the EF. For example, thriving fungi have been shown to increase the structural rigidity of soils.^17^ Fungi are also known to impact recycling of carbon by degrading polyaromatic hydrocarbons (PAHs).^18^ The capability of microorganisms to grow in EFs and cause aggregates of large particulates requires a study dedicated to quantifying how these microorganisms affect overall EF growth and maturation.

## Experimental Section

### Sample Collection

EFs were collected on single-side-polished silicon wafer substrates (Silicon Valley Microelectronics). Before exposure, wafers were cleaned using a combination of UV-ozone (Jelight Model 42) exposure and rinsing with optical grade ethanol (KOPTEC). The wafers were transported to and from sampling locations using cleaned Petri dishes. Exposure times for the silicon substrate to accumulate environmental films were either 2 or 12 months, as noted for each. The 2 month samples were exposed during February and March in 2019. The 12 month samples were placed from October 2018 to October 2019. The sampling sites were at the University of Iowa chemistry building (CB) (Lat: 41.66408, Lon: −91.53682) and Iowa City Park (CP) (Lat: 41.67632, Lon: −91.54332). It should be noted that both sites are close to the Iowa River which could affect microorganism profiles. Ecosystems near rivers and other bodies of water, known as riparian areas, benefit from terrestrial and aquatic nutrients creating more fertile soils than isolated dry areas.^19^ The richness of the nutrients in this ecosystem increases fungal activity, which could mean that fungi have a higher presence in these areas than in cities; in different ecosystems, this could account for discrepancies here versus other sampling sites. During sampling, the wafers are supported on an aluminum sampling station which has been described in prior work.^2^

### Characterization Methods

Samples were imaged using a Hitachi S 4800 scanning electron microscope with an IXRF X-ray detector to allow energy dispersive X-ray spectroscopy (EDS). Electron accelerating voltages of 15–25 keV were used for these analyses with a current of 10 μA. A live time of 100 s was used to gather enough data to ensure high signal-to-noise ratio.

### Bright Field Microscopy

Images were taken using an Olympus Stereoscope SZX12 microscope. The magnification was 50× with 100 ms exposure time. The imaging camera has a rectangular array of 1600 × 1200 pixels. An ImageJ macro was used to identify and tabulate the number of fungi per unit area and surface coverage from collected images. The details of the macro are listed in Figure S1.

## Results and Discussion

### Quantifying the Impact of the fungi

**Figure 1:**
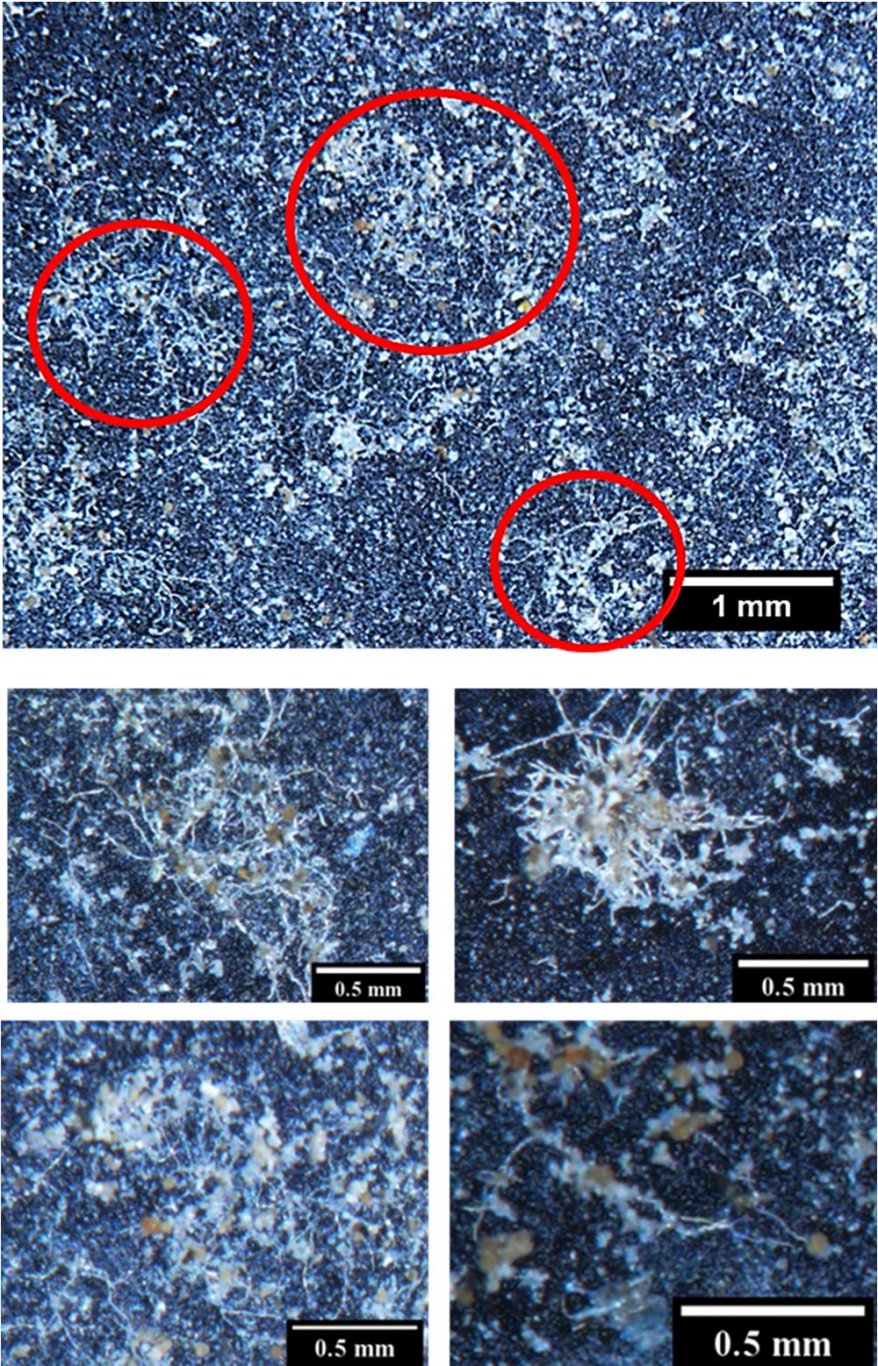
Optical microscopy of a 12 month environmental film. Red circles highlight selected centers of fungi growth with aggregation and large reach across the surface (top). Bottom images show the large-particle aggregating capacity of the fungi (bottom four images).

Figure 1, top is an image showing significant microorganism clusters and hypha (bright white, branched morphologies) that form an extended network across the surface. Red circles highlight areas with particularly high concentrations of microorganisms. Over 12 similar images, we found that there is an average of 18 ± 2 fungi colonies/cm^2^ (defined as a growth that has connected hyphae but is resolvable from other surface features) which constitutes 14 ± 1% surface coverage. Further studies must be done to classify how the proximity to point sources, such as rivers or streams, might affect this metric. The four bottom images of Figure 1 suggest that large particle aggregation is also prevalent on the microorganism clusters. These images are enlarged here, but acquired at the same magnification, to show the significant particle co-locality with the hypha. While there are particles distributed over most of the EF surface, the largest particles are primarily localized to the microorganism hypha. Smaller particles are more homogeneously dispersed and not necessarily collocated with the hypha. Studying the composition of the hyphae and entrained aggregates is a complex but important problem. To reduce sample complexity while maintaining the ability to report chemical species associated with the fungi, we chose to examine films collected for *2* months as opposed to 12 months (as shown in Figure 1).

### Mapping Elemental Species Adjacent to Fungal Colonies

**Figure 2:**
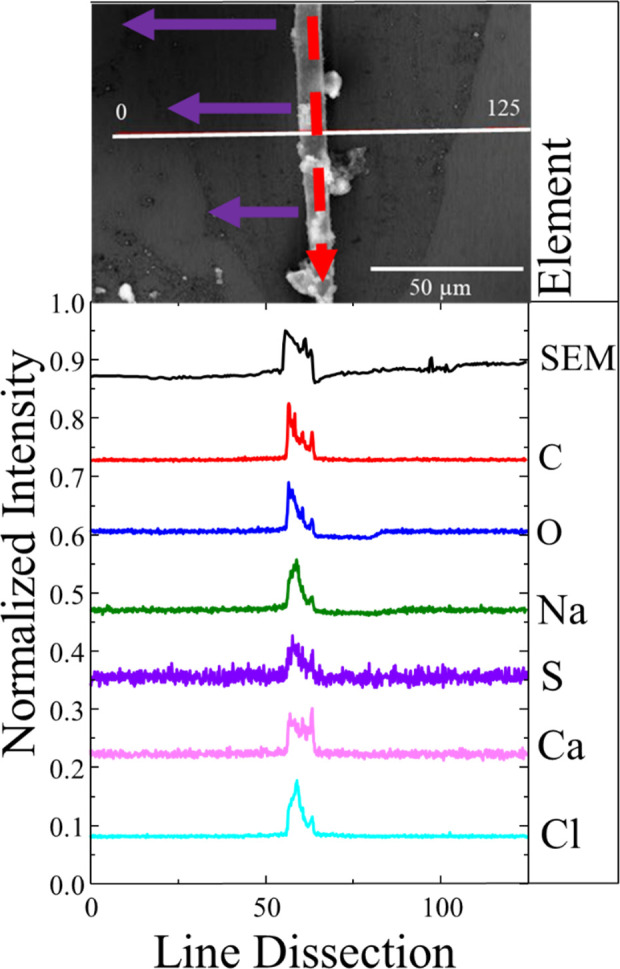
SEM image (top) of fungi in an environmental film. Red arrow shows the direction of the fungi growth, and purple arrows show the extent of the nutrient pool. EDS elemental intensities along the horizontal white line are shown below with most intensity localized near the hyphae.

The top of Figure 2 shows an SEM image of fungal growth on a 2 month EF surface. The horizontal white line represents a line dissection by EDS, which is shown in the bottom of Figure 2. The dashed red arrow indicates the hyphae’s suggested growth direction. Extending from the hyphae, we note a substantial change in contrast in the SEM images which we assign to a “nutrient pool” that extends several tens of micrometers in radius. Figure S2 is a false color representation of this SEM image to delineate the hyphae (purple), nutrient pool (green), and background EF (yellow) areas more clearly. The dark color of the pool suggests increased conductivity or electron density of the local material versus the adjacent surface areas. This pool represents the liquid volume over which fungi will excrete extracellular enzymes to digest large molecules and exchange nutrients/ions in a local solution.^20^ The pool likely also contains nutrient sources and waste products from adjacent, symbiotic microorganisms such as bacteria.^9^ The SEM images show that the distal extent of the pool varies with respect to the fungi’s long axis. This is likely due to the growth mechanism of the fungi: as it grows from top to bottom in Figure 2, the affected pool will be smaller near the newer (younger) tip of the hyphae. In other words, the pool expands laterally as the fungi grow, seen in a smaller spread near the hyphae tip. The growth pattern of the pool suggests that longer residence times of the microorganism will have larger impacts on the EF, easily doubling (or more) the area of simple surface coverage estimated above.^11^ While the contrast in the SEM image suggests the presence of different elements of interest, like those associated with the biotic activity, the SEM-EDS traces shown in Figure 2, bottom do not clearly identify the pool boundaries. EDS does show particulates concentrated near the hyphae, likely due to adsorption or vacuum induced crystallization of nearby aqueous brine during SEM imaging.^21^ This suggests that SEM contrast representing the nutrient pool might arise from accumulated water near the hypha. Further studies are needed to understand the chemical and biological causes of these domains.

The EDS plots show uniformly higher elemental intensity localized to the hyphae. We expect higher amounts of carbon in this area because the of the carbon-based fungal hyphae.^22^ The spatial overlap of carbon, oxygen, sodium, chlorine, and sulfur indicates that these species are also highly concentrated to the central fungi. We attribute additional signals represented by intensity spikes in these traces to adsorbed particulates. This “fine structure” of peaks in the carbon trace suggests that particles, likely oxalate, adsorbed to the fungi also contribute to the carbon signal. The calcium intensity trace varies uniformly across the fungi. In comparison, the carbon and oxygen have peak intensity values corresponding to the hyphae’s location which is at a ratio of ∼2:1 oxygen:carbon. This is evidence of oxalate/oxalic acid^23^ which we attribute to either (1) deposited particulate from the atmosphere or (2) previously aqueous phase minerals which have nucleated onto the hyphae in the high vacuum conditions of the SEM chamber. Several such particles are clearly present in the SEM image. Line traces for other elements relevant to biotic activity are included in Figure 2. Amounts of these elements are proportional to EDS intensity which allows suggestions of molecular formulas, e.g., a 1:1 ratio for Na and Cl could indicate sodium chloride. We note that sodium and chloride have been found in EFs and are attributed to the deposition of aerosolized road salts.^24,25^ This could be a possible nutrient source for fungi, but making this assignment would require more detailed studies of the nutrient pool and profiling of fungal colonies. Figure S3 shows additional SEM images of fungi with adsorbed particulate, acquired from the CP (left) and CB (right) sampling locations. Additional EDS line dissections are provided in Figure S4 (CB) and Figure S5 (CP) to emphasize the reproducibility of these results across sampling sites.

## Conclusion

The impacts of fungi are shown throughout literature, including the fungi’s capacity to aggregate soil and recycle carbon from various sources including polluting species such as PAHs.^17,18^ The work we present here suggests that the fungi present in environmental films may also exhibit important behaviors, on long time scales, as they modify environmental film morphology and chemistry. We have conducted an initial study of fungi in environmental films collected in the Iowa City area, near the Iowa River. Bulk properties of the films (passively sampled for 1 year) showed high biotic specific surface coverage (ca. 14%) with particulate aggregation associated the fungi colonies. This is important because it shows the films will have similar behaviors to fungi existing in the soil. To report chemical impacts, shorter exposure (2 month) environmental films were analyzed. There is also evidence of a nutrient pool propagating laterally across the EF, orthogonal to the hyphae growth direction over several tens of micrometers. This interaction may provide insights to the ionic losses reported previously,^26^ and it will improve our overall understanding of environmental film behaviors. Further studies will done to report more details regarding how the fungi will influence EF chemistry and morphology, particularly over larger geographic areas and varying seasons.

## Supporting Information Available

ImageJ code and sample image highlighting fungal growth, illustration showing the nutrient pool and central hypha, SEM images showing accumulated species on the hypha, and replicates of the line dissections (PDF)
