# Supplementary material for: Association of Chemical Aggregates and Fungal Moieties Affecting Native Environmental Films
Source: ACS Environ Au. 2022 Apr 14;2(4):310–3. doi: 10.1021/acsenvironau.2c00004 (PMC10125300; doi:10.1021/acsenvironau.2c00004)
Supplement: Supplementary file 1 — vg2c00004_si_001.pdf [file vg2c00004_si_001.pdf]

Supporting Information for: Association of chemical aggregates and fungal moieties affecting native environmental films

Authors:

1) Jessica L DeYoung, University of Iowa, Iowa City ORCID: 0000-0001-7484-4968

2) Scott K. Shaw*, University of Iowa, Iowa City ORCID: 0000-0003-3767-3236

*corresponding author

Table of Contents

Figure S1: ImageJ macro code used to calculate the density of fungi/cm^2 with the image outlines showing the results of the analysis…………2

Figure S2: the image from Figure 2 showing the designations for fungal hyphae, the nutrient pool, and the underlying surface…………3

Figure S3: SEM images showing particulate accumulation along the hyphae of the structures, the tips of the structures, and an SEM image showing other evidence of the nutrient pool…………4

Figure S4: A representative analysis of the line dissection from the CB location…………5

Figure S5: Line dissections from two different points showing chemical changes long the growth of the hyphae…………6

Figure S1: ImageJ macro code used to calculate the density of fungi/cm^2 with the image outlines showing the results of the analysis

The ImageJ Program for identifying the concentration (fungi/cm2) and surface coverage:
run("Subtract Background...", "rolling=50"); %corrects uneven lighting
run("Enhance Contrast...", "saturated=10"); %enhances contrast of colors to ensure detection of more dark particles
setOption("BlackBackground", false); %ensures that the background (silicon wafer) will be set to black not inverted
run("Convert to Mask"); %Converting the image to binary for particle analysis
run("Analyze Particles...", " circularity=0.00-2 show=Outlines clear summarize"); %analyzing particulate with low circularities showing the outlines to ensure we are counting just biotic activity and associated particulate.

Resulting Images:

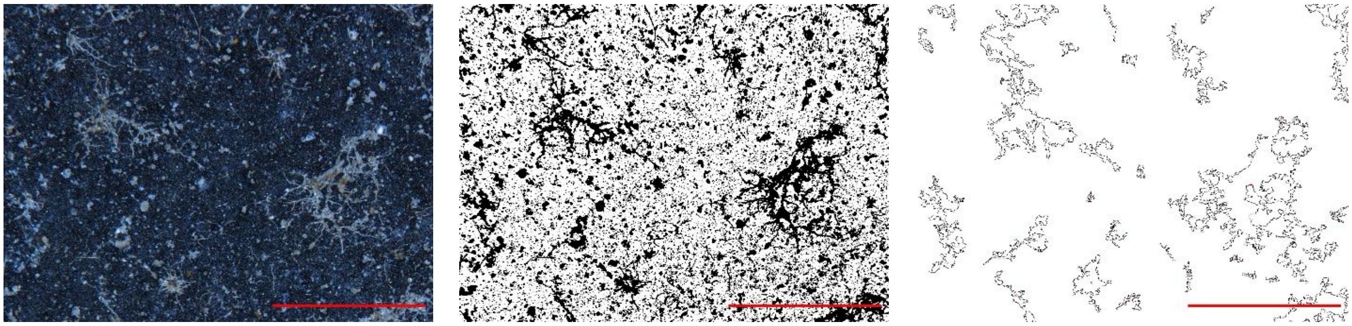

Figure SI:1 The bright field image (left), binary image (middle), and image showing outlines of analyzed particulate (right) that the code produces. The scalebar is 0.5 mm in each.

Figure S2: the image from Figure 2 showing the designations for fungal hyphae, the nutrient pool, and the underlying surface

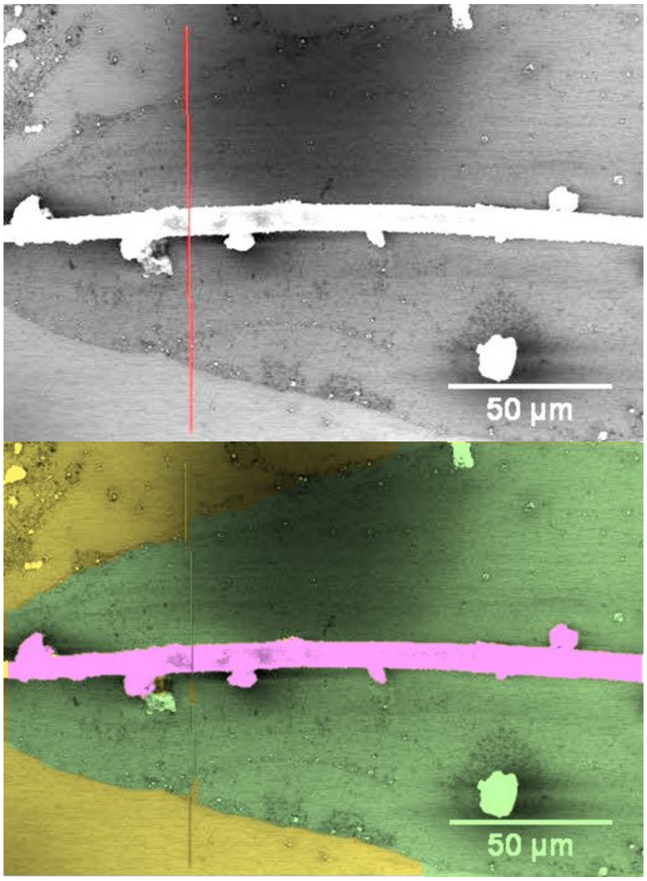

Figure S2:An SEM image suggesting the existence of the nutrient pool with the EDS line dissection (top). The bottom image shows the areas in for clarification of nomenclature. The yellow is the surface, green is the "nutrient pool", and the pink is the hyphae of the central microorganism.

Figure S3: SEM images showing particulate accumulation along the hyphae of the structures, the tips of the structures.

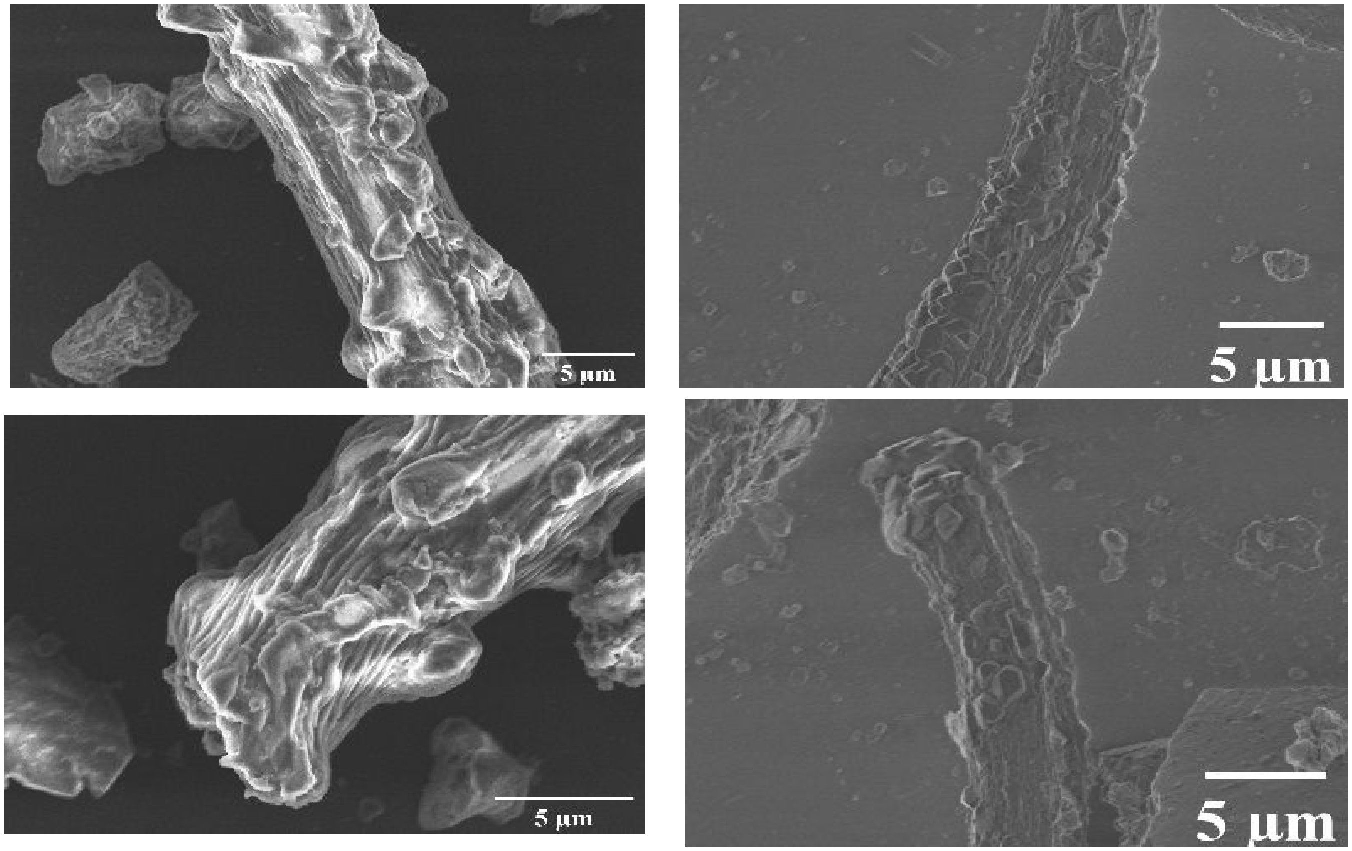

Figure S3: samples from city park (left) and chemistry building (right) showing adsorbed particulate to the central fungi.

Figure S4: A representative analysis of the line dissection from the CB location

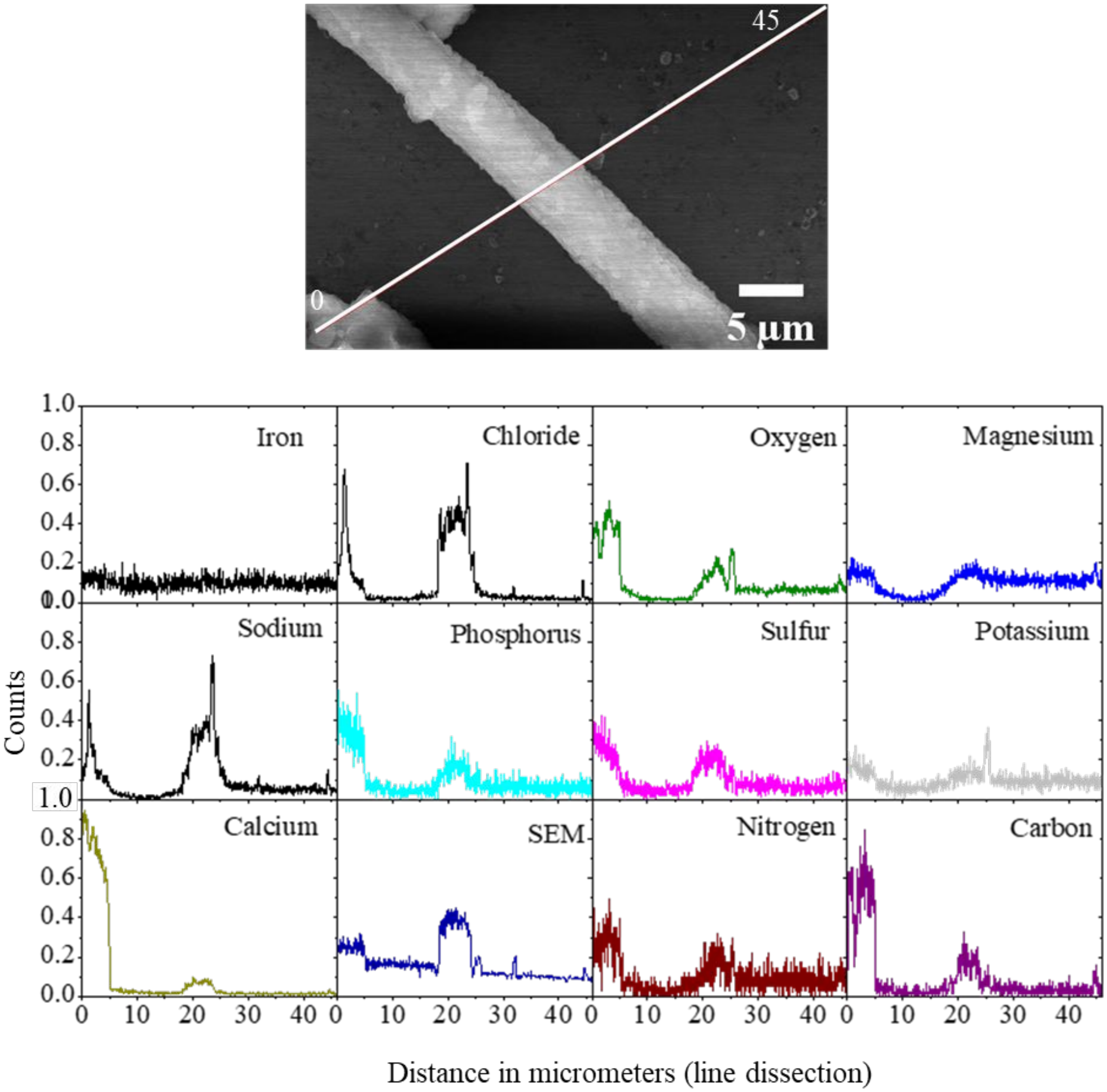

Figure S4: an SEM image and elemental line-traces showing localization to the fungal hyphae. Sample taken from CB site.

Figure S5: Line dissections from two different points showing chemical changes long the growth of the hyphae

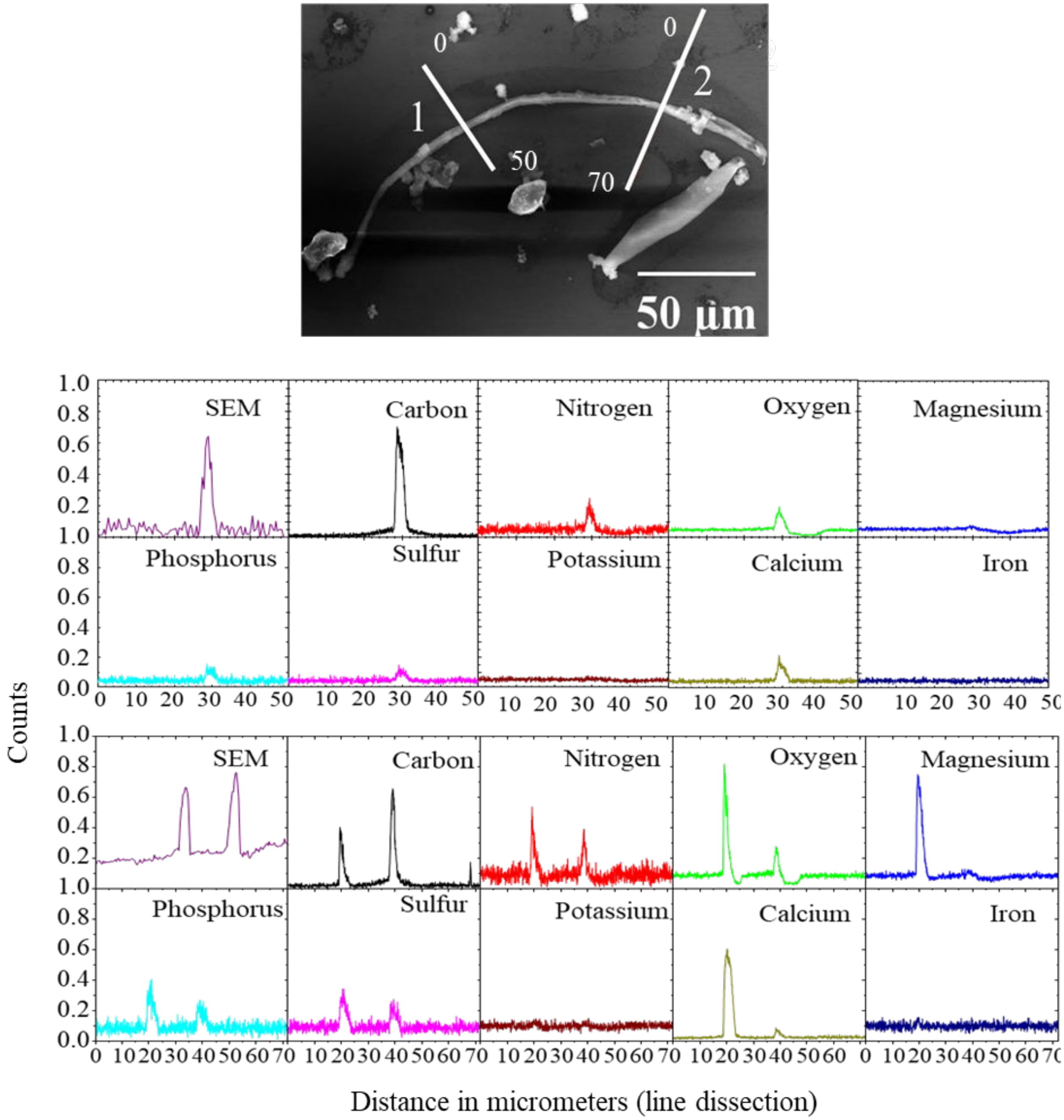

Figure S5: Elemental dissections elemental line-traces showing localization to the fungal hyphae as the microorganism grows. Elements of interest remain localized to the hyphae. Sample taken from CP site.
